# The relationship between body mass index and sleep in women with risk factors for gestational diabetes mellitus

**DOI:** 10.1002/osp4.689

**Published:** 2023-06-17

**Authors:** Pamela Acosta Reyes, Jincy Immanuel, William M. Hague, Helena Teede, Emily Hibbert, Christopher J. Nolan, Michael J. Peek, Vincent Wong, Jeffrey R. Flack, Mark McLean, Raiyomand Dalal, Jürgen Harreiter, Alexandra Kautzky–Willer, Rohit Rajagopal, Arianne Sweeting, Glynis P. Ross, Ngai Wah Cheung, David Simmons

**Affiliations:** ^1^ Macarthur Clinical School Western Sydney University Sydney New South Wales Australia; ^2^ Robinson Research Institute The University of Adelaide Adelaide South Australia Australia; ^3^ Monash University Melbourne Victoria Australia; ^4^ Faculty of Medicine and Health University of Sydney Sydney New South Wales Australia; ^5^ Department of Endocrinology Nepean Hospital Sydney New South Wales Australia; ^6^ Department of Endocrinology The Canberra Hospital Canberra Australian Capital Territory Australia; ^7^ School of Medicine and Psychology College of Health and Medicine Australian National University Canberra Australian Capital Territory Australia; ^8^ Liverpool Hospital Sydney New South Wales Australia; ^9^ Bankstown‐Lidcombe Hospital Sydney New South Wales Australia; ^10^ Blacktown Hospital Sydney New South Wales Australia; ^11^ Campbelltown Hospital Campbelltown New South Wales Australia; ^12^ Department of Medicine III Division of Endocrinology Gender Medicine Unit Medical University of Vienna Vienna Austria; ^13^ Department of Endocrinology Royal Prince Alfred Hospital Sydney New South Wales Australia; ^14^ Department of Diabetes & Endocrinology Westmead Hospital Sydney New South Wales Australia

**Keywords:** GDM, obesity, pregnancy, sleep, snore

## Abstract

**Background:**

Both obesity and sleep disorders are common among women during pregnancy. Although prior research has identified a relationship between obesity and sleep disorders, those findings are from women later in pregnancy.

**Objective:**

To explore the relationships between self‐reported sleep duration, insufficient sleep and snoring with body mass index (BMI) among multiethnic women at risk of gestational diabetes mellitus (GDM)in early pregnancy.

**Methods:**

Cross‐sectional study of baseline data from women at risk of GDM enrolled in the Treatment of BOoking Gestational diabetes Mellitus (TOBOGM) multicentre trial across 12 Australian/Austrian sites. Participants completed a questionnaire before 20 weeks’ gestation to evaluate sleep. BMI <25 kg/m^2^ served as the reference group in multivariable logistic regression.

**Results:**

Among the 2865 women included, the prevalence of overweight and obesity classes I‐III was 28%, 19%, 11% and 12%, respectively. There was no relationship between sleep duration and BMI. The risk of insufficient sleep >5 days/month was higher in class II and class III obesity (1.38 (1.03–1.85) and 1.34 (1.01–1.80), respectively), and the risk of snoring increased as BMI increased (1.59 (1.25–2.02), 2.68 (2.07–3.48), 4.35 (3.21–5.88) to 4.96 (3.65–6.74), respectively)).

**Conclusions:**

Obesity is associated with insufficient sleep among pregnant women at risk of GDM. Snoring is more prevalent with increasing BMI.

## 1 INTRODUCTION

The most common metabolic disorder in pregnant women is diabetes, which complicates 1 in 6 (16.8%) pregnancies, with gestational diabetes mellitus (GDM) making up 86% of the total.^1^ Obesity during pregnancy is the major risk factor for GDM,^2^ and there is a greater risk of preeclampsia, caesarean section and maternal morbidity when obesity and GDM occur together than separately.^3^

Many women develop sleep disturbances during pregnancy,^4^, ^5^ particularly in late pregnancy.^6^ These include difficulty falling asleep, frequent night waking and restless sleep by the end of pregnancy.^7^ Pregnant women experience a decrease in restorative deep sleep as early as weeks 11 and 12 of gestation,^8^ and only 54% of pregnant women sleep for at least 8 h uninterrupted in the last trimester.^9^ Many factors contribute to sleep disturbance during pregnancy.^9^, ^10^, ^11^ Excess gestational weight gain is experienced by 64% of women who are overweight or obese and is associated with decreased sleep hours and sleep quality during the late stages of pregnancy.^12^, ^13^

Obesity has been linked with a high risk of sleep disturbance.^14^, ^15^ Some studies have revealed a bidirectional relationship between obesity and sleep quality.^16^ Sleep disturbances interfere with endocrine pathways that regulate energy balance, stimulating weight gain and obesity.^17^ Some studies,^18^, ^19^ but not others,^20^, ^21^ have suggested that weight reduction has a positive impact on obstructive sleep apnea (OSA) outside of pregnancy.^22^ One study suggested that awareness of basic nutrition and customized diet plans are associated with improved sleep and reduced snoring.^23^ The occurrence of sleep‐disordered breathing is higher during pregnancy compared with non‐pregnant individuals,^24^ yet few intervention studies have been reported.^25^, ^26^ A recent meta‐analysis^27^ showed an increased risk of snoring, short sleep duration and poor sleep quality among pregnant women with high BMI; however, most of the studies included were conducted late in gestation.

Therefore, a cross‐sectional study was conducted, using data from a large multicentre randomized control trial (RCT), among pregnant women at risk of GDM at an early stage of pregnancy. The aims of this study were to compare the amount of sleep, insufficient sleep and self‐reported snoring in different BMI groups within a multiethnic cohort of pregnant women at risk of GDM participating in the TOBOGM study.^28^ Limited nutritional data were collected, and BMI was therefore used as a proxy for all its contributing factors.

## 2 METHODS

A cross‐sectional cohort study of women at baseline enrolled in the TOBOGM multicentre RCT was performed. This trial is investigating the benefits and adverse effects of the early treatment of GDM diagnosed before 20 weeks’ gestation according to the International Association of the Diabetes and Pregnancy Study Groups (IADPSG) criteria.^28^, ^29^ Women at risk of GDM recruited prior to the coronavirus (COVID19) pandemic to TOBOGM from the antenatal clinics of the participating hospitals between June 2017 and March 2020 were included. Women were eligible if they were aged ≥18 years, were carrying a singleton pregnancy between 4 and 19^+6^ weeks’ gestation, had a risk factor (e.g., being overweight or obese, advanced maternal age, multiparous, family history of type 2 diabetes mellitus, non‐European descent) for GDM and had provided written informed consent.^29^ Women with pre‐existing diabetes, with overt diabetes or fasting blood glucose ≥6.1 mmol/L, or with major medical disorders were excluded. All enrolled women completed a booking demographic questionnaire that included the ethnicity that the participant identified with, and medical and obstetric history. Height and weight were extracted from medical records. After calculation, BMI was categorized into five groups: <25.0 kg/m^2^, overweight (25.0–29.9 kg/m^2^), obesity class I (30.0–34.9 kg/m^2^), obesity class II (35.0–39.9 kg/m^2^), and obesity class III (≥40.0 kg/m^2^), based on the World Health Organization (WHO) classification.^30^

Ethnicity was divided into two main groups (European (of European descent) and non‐European) for the statistical analysis due to the lower number of participants in any one non‐European group.

Sleep parameters were collected at recruitment using a written questionnaire adapted from the Pittsburgh Sleep Quality Index (PSQI) questionnaire^31^ by shortening to reduce participant questionnaire burden. Sleep duration, insufficient sleep and snoring were assessed, using the following questions: “How many hours do you sleep per 24 h on average?”, “How many days in the last month have you had the feeling of insufficient sleep?”, and “How many days per week do you snore/are you told you snore?”, respectively.

The main outcomes of the study among pregnant women in early pregnancy were decreased sleep duration less than 8 h per day (selected to allow dichotomization of the variable around the median), insufficient sleep more than 5 days per month (selected as the population median) and the presence of snoring. Blood glucose results, GDM status and pregnancy outcomes were kept separate from the baseline dataset in accordance with the blinded nature of the trial and were not included in this study. The study was approved by the South Western Sydney Local Health District Ethics committee (reference 15/LPOOL/551). In Austria, the study was approved by the Ethics committee of the Medical University of Vienna (1337/2016). The study was registered with the Australian New Zealand Clinical Trial Registry (ACTRN12616000924459).

### 2.1 Statistical analysis

Categorical variables have been described using frequencies and percentages, and continuous variables by means and standard deviations. Maternal characteristics between BMI groups have been compared, using one‐way analysis of variance (ANOVA) for continuous variables and the Pearson's *χ* ^2^ test for binary data. Multivariable logistic regression models were performed to evaluate the relationship between BMI categories and sleep parameters, adjusting for potential confounders, including age, ethnicity, smoking, employment status, gestational age, parity, married/living together, alcohol consumption before pregnancy, and family history of diabetes. All statistical analyses were conducted using SPSS software (IBM SPSS Statistics, Version 25.0). All tests were two‐tailed and a *p*‐value <0.05 was considered significant.

## 3 RESULTS

### 3.1 Comparisons of maternal characteristics between BMI groups

Of the 2897 women enrolled in the timeframe of this sub‐study, 2865 had a BMI recorded before 20 weeks’ gestation and were therefore included in the analysis. The mean age of the women was 31.3 ± 5.1 years. The mean BMI was 29.9 ± 7.6 kg/m^2^ at booking (between 4 and 19^+6^ weeks' gestation). The majority had a European (37.7%), Middle Eastern (7.9%) or South Asian (26.9%) background (Table 1). The prevalence of overweight was 28.1% (*n* = 806) and the overall prevalence of obesity was 41.5% (*n* = 1202) (class I obesity, 18.7% (*n* = 537), class II obesity, 11.4% (*n* = 326), and class III obesity 11.8% (*n* = 339)). The women reported sleeping 7.8 ± 1.4 h per day and 7.8 ± 8.9 days of insufficient sleep per month (median 5 days). The reported prevalence of snoring was 36.6%.

**TABLE 1:** Comparisons of maternal characteristics among BMI groups.

Characteristics	All		<25 kg/m^2^		25.0–29.9 kg/m^2^		30.0–34.9 kg/m^2^		35.0–39.9 kg/m^2^		≥40.0 kg/m^2^		*p* value
	*N* = 2865		*N* = 857 (29.9%)		*N* = 806 (28.1%)		*N* = 537 (18.7%)		*N* = 326 (11.4%)		*N* = 339 (11.8%)		
	N	Mean ± SD/n (%)	N	Mean ± SD/n (%)	N	Mean ± SD/n (%)	N	Mean ± SD/n (%)	N	Mean ± SD/n (%)	N	Mean ± SD/n (%)	
Age (years)	2865	31.3 ± 5.1	857	31.7 ± 4.9	806	31.8 ± 5.1	537	31.2 ± 5.4	326	30.6 ± 5.1	339	30.3 ± 4.9	<0.001
Height (cm)	2865	162.8 ± 6.9	857	162.0 ± 6.7	806	161.4 ± 6.6	537	163.4 ± 6.8	326	164.4 ± 7.1	339	165.5 ± 6.4	<0.001
Booking weight (kg)	2865	79.7 ± 22.7	857	58.7 ± 6.8	806	71.1 ± 7.0	537	86.1 ± 8.5	326	100.7 ± 9.2	339	123.3 ± 15.0	<0.001
Self‐reported pre‐pregnancy weight (kg)	2839	76.7 ± 22.5	853	56.6 ± 8.0	795	68.0 ± 7.7	535	83.1 ± 9.8	322	97.4 ± 10.7	334	118.8 ± 16.0	<0.001
Gestation at booking (weeks)	2865	14.7 ± 2.5	857	14.7 ± 2.5	806	14.7 ± 2.5	537	14.8 ± 2.5	326	14.8 ± 2.3	339	14.8 ± 2.4	0.88
Ethnicity	2857		854		804		536		325		338		
European		1097 (37.7)		203 (23.8)		225 (28.0)		243 (45.3)		188 (57.8)		220 (65.1)	<0.001
Middle Eastern		227 (7.9)		56 (24.7)		81 (35.7)		40(17.6)		26 (11.4)		20 (8.8)	
South Asian		769 (26.9)		304 (39.5)		298 (38.7)		116 (15.0)		28 (3.6)		11 (1.4)	
Southeast Asian/East Asian		425 (14.8)		241 (28.2)		123 (15.3)		43 (8.0)		12 (3.7)		6 (1.8)	
Other		374 (13.1)		50 (5.9)		78 (9.7)		94 (17.5)		71 (21.8)		81 (24.0)	
Married/living together	2842	2665 (93.8)	849	816 (96.1)	800	764 (95.5)	532	494 (92.9)	324	300 (92.6)	337	291 (86.4)	<0.001
Working full‐time	2865	1063 (37.1)	857	324 (37.8)	806	320 (39.7)	537	194 (36.1)	326	130 (39.9)	339	95 (28.0)	0.003
Working part‐time	2865	699 (24.4)	857	241 (28.1)	806	183 (22.7))	537	124 (23.1)	326	74 (22.7)	339	77 (22.7)	0.056
Gravidity	2865	2.6 ± 1.7	857	2.3 ± 1.5	806	2.6 ± 1.7	537	2.9 ± 1.9	326	2.7 ± 1.6	339	2.9 ± 1.8	<0.001
Parity	2865	1.0 ± 1.1	857	0.8 ± 1.0	805	1.0 ± 1.1	537	1.2 ± 1.2	326	1.0 ± 1.1	339	1.2 ± 1.2	<0.001
Current smoking	2713	144 (5.3)	826	28 (3.4)	765	32 (4.2)	504	30 (6.0)	301	18 (6.0)	317	36 (11.4)	<0.001
Alcohol consumption before pregnancy	2679	1068 (39.9)	816	291 (35.7)	754	275 (36.5)	498	220 (44.2)	297	132 (44.4)	314	150 (47.8)	<0.001
Prior GDM	2044	432 (21.1)	532	115 (21.6)	601	129 (21.5)	405	90 (22.2)	243	37 (15.2)	263	61 (23.2)	0.19
Family history of diabetes	2704	1193 (44.1)	819	351 (42.9)	763	393 (51.5)	502	214 (42.6)	300	119 (39.7)	320	116 (36.3)	<0.001
Self‐reported PCOS	2854	518 (18.1)	853	129 (15.1)	802	138 (17.2)	534	104 (19.5)	326	75 (23.0)	339	72 (21.2)	0.003
Sleep characteristics													
Less than 8 h of sleep per day	2682	1062 (39.6)	819	297 (36.3)	761	287 (37.7)	498	217 (43.6)	293	123 (42.0)	311	138 (44.4)	0.02
Insufficient sleep more than 5 days/month	2608	1084 (41.6)	787	278 (35.3)	742	281 (37.9)	489	214 (43.8)	290	149 (51.4)	300	162 (54.0)	<0.001
Snoring	2540	921 (36.3)	765	175 (22.9)	704	221 (31.4)	479	204 (42.6)	290	155 (53.4)	302	166 (55.0)	<0.001

Abbreviations: BMI, body mass index; GDM, Gestational diabetes mellitus; PCOS, Polycystic ovary syndrome.

Table 1 shows the comparison of baseline and sleep characteristics between BMI groups. The characteristics of the BMI groups differed significantly, except for gestation at booking and prior GDM status. The proportion of women sleeping fewer than 8 h a day varied across groups, with higher prevalence (44.4%) among the class III obesity group (*p* = 0.02). The proportions of women reporting insufficient sleep more than 5 days per month increased across the BMI range from 37.9% to 43.8%, 51.4%, and 54.0% in overweight to class I, class II, and class III obesity, respectively (*p* < 0.001). The trend was similar for snoring (31.4%, 42.6%, 53.4%, and 55.0% respectively, *p* < 0.001).

### 3.2 Association between BMI categories and sleep parameters according to ethnic group

European women were more likely than non‐European women to report sleeping <8 h and experience >5 days/month of insufficient sleep among women with BMI <25.0 kg/m^2^. Within high BMI groups, there were no differences in sleep duration and snoring comparing women of European and non‐European descent, but European women had a higher rate of experiencing insufficient sleep >5 days per month in all overweight and obese BMI groups (Table 2).

**TABLE 2:** Association between BMI categories and sleep parameters according to ethnic groups (European/non‐European).

Sleep parameters	<25.0 kg/m^2^ (*N* = 854)			25.0–29.9 kg/m^2^ (*N* = 804)			30.0–34.9 kg/m^2^ (*N* = 536)			35.0–39.9 kg/m^2^ (*N* = 325)			≥40 kg/m^2^ (*N* = 338)		
	Non‐European	European	*p* value	Non‐European	European	*p* value	Non‐European	European	*p* value	Non‐European	European	*p* value	Non‐European	European	*p* value
	*N* = 651 (76.2%)	*N* = 203 (23.8%)		*N* = 579 (72.0%)	*N* = 225 (28%)		*N* = 293 (54.7%)	*N* = 243 (45.3%)		*N* = 137 (42.2%)	*N* = 188 (57.8%)		*N* = 118 (34.9%)	*N* = 220 (65.1%)	
Less than 8 h of sleep per day (*N* = 2679)	214/624 (34.3)	83/194 (42.8)	0.03	199/552 (36.1)	88/208 (42.3)	0.13	116/276 (42.0)	100/221 (45.2)	0.52	53/122 (43.4)	70/171 (40.9)	0.72	44/107 (41.1)	94/204 (46.1)	0.47
Insufficient sleep >5 days per month (*N* = 2604)	180/596 (30.2)	97/189 (51.3)	<0.001	168/534 (31.5)	113/207 (54.6)	<0.001	83/269 (30.9)	130/219(59.4)	<0.001	51/117 (43.6)	98/173 (56.6)	0.03	40/98 (40.8)	122/202 (60.4)	0.002
Snoring (*N* = 2536)	133/574 (23.2)	42/189 (22.2)	0.84	167/506 (33.0)	54/197 (27.4)	0.18	105/260 (40.4)	98/218 (45.0)	0.35	67/121 (55.4)	88/169 (52.1)	0.63	60/102 (58.8)	106/200 (53.0)	0.39

*Note*: The numerator is the total of participants that present the sleep parameter, and the denominator is the total of participants in each BMI category that answered the questionnaire.

### 3.3 Risk of developing sleep disorders among different BMI groups

Table 3 shows the risk of sleep disturbances in each BMI group. There was not an association between the control group and higher BMI. Class II and III obesity were associated with increased risk of insufficient sleep (aOR (95% CI) 1.38(1.03–1.85), and 1.34 (1.01–1.80)), respectively. The risk of snoring increased with an increased BMI and persisted after adjusting for potential confounders, including full‐time employment (as long working hours can cause sleep disturbances) (Table 3).

**TABLE 3:** Adjusted and unadjusted risk of developing sleep disorders among different BMI groups.

Self‐reported sleep parameters	Unadjusted OR (95% CI), *p* value				Adjusted OR (95% CI), *p* value			
	25.0–29.9 kg/m^2^	30.0–34.9 kg/m^2^	35.0–39.9 kg/m^2^	≥40.0 kg/m^2^	25.0–29.9 kg/m^2^	30.0–34.9 kg/m^2^	35.0–39.9 kg/m^2^	≥40.0 kg/m^2^
	*N* = 806	*N* = 537	*N* = 326	*N* = 339	*N* = 806	*N* = 537	*N* = 326	*N* = 339
Less than 8 h of sleep per day	1.06 (0.87–1.31), 0.55	1.36 (1.08–1.70), 0.008	1.27 (0.97–1.67), 0.08	1.40 (1.07–1.83), 0.01	1.03 (0.83–1.27), 0.80	1.26 (0.99–1.60), 0.06	1.16 (0.87–1.55), 0.31	1.26 (0.95–1.68), 0.11
Insufficient sleep more than 5 days/month	1.12 (0.91–1.38), 0.30	1.42 (1.13–1.80), 0.003	1.94 (1.47–2.54), <0.001	2.15 (1.64–2.82), <0.001	1.02 (0.82–1.27), 0.89	1.12 (0.87–1.43), 0.38	1.38 (1.03–1.85), 0.03	1.34 (1.001–1.80), 0.049
Snoring	1.54 (1.22–1.95), <0.001	2.50 (1.95–3.20), <0.001	3.87 (2.91–5.15), <0.001	4.12 (3.10–5.46), <0.001	1.59 (1.25–2.02), <0.001	2.68 (2.07–3.48), <0.001	4.35 (3.21–5.88), <0.001	4.96 (3.65–6.74), <0.001

*Note*: Reference group = BMI <25 kg/m^2^; Results were adjusted for age, ethnicity as European/non‐European descent, smoking, gestation at booking, parity, full‐time employment, married/living together, alcohol consumption before pregnancy, and family history of diabetes.

## 4 DISCUSSION

This study identified a strong positive relationship between BMI category and indices of sleep disturbance early in pregnancy in a large multiethnic cohort of pregnant women at risk of GDM. Findings, after adjusting for relevant confounders, suggest an increased risk of insufficient sleep and snoring among women with obesity in early pregnancy. Insufficient sleep was reported more often by European women than non‐European women. This study also found that the risk of snoring increased as maternal BMI increased, even after adjusting for relevant confounders.

Study results are consistent with an earlier study reporting that women with obesity have a 1.6‐fold higher likelihood of poor sleep quality than lean women.^15^ Findings are also in agreement with the Hill et al.^14^ study that found higher rates of poor sleep quality among women with a BMI >25 kg/m^2^ in early pregnancy. Conlon et al.^32^ conducted a study among overweight or obese pregnant women, exploring changes in sleep through pregnancy using the PSQI score. Early in pregnancy, 47% of these overweight and obese women had a PSQI score >5, indicative of “poor sleep”. Also, a study conducted by Schwab et al.^33^ have conclude that there is a relationship between patients with obesity, including a larger tongue, and obstructive sleep apnea. On the other hand, Nicoli et al.^34^ assessed sleep quality and nocturnal sleep duration and reported that sleep disturbances are not a risk factor for GDM in pregnant women at high risk. According to Castellucci et al.,^35^ many studies have demonstrated a positive and two‐way association between sleep disturbances and obesity.^36^

To address these variations in findings, a meta‐analysis of 46 observational studies has been published, which included articles from 16 different countries, assessing different sleep parameters objectively and subjectively.^27^ Findings were in line with this study, but this is a large multicentre study with participants from different nationalities with risk factors for GDM. Additionally, a recent systematic review showed that women with overweight and obesity before pregnancy experienced poor sleep and had a higher risk of obstetric complications and cardiometabolic diseases after pregnancy.^37^ The mechanisms behind this relationship remain unclear and while there may be a direct physical effect of obesity, indirect mechanisms through food choices and eating patterns or limited physical activity warrant investigation.

An issue for this study was over which BMI cut‐offs to use within this multiethnic cohort. According to Fattah et al.,^38^ weight and body composition in pregnant women during first trimester are unchanged from pre‐pregnancy. Therefore, this study used the BMI classification for non‐pregnant adults unchanged. An alternative BMI classification has been proposed, beyond the WHO classification used in this paper, for Asian populations.^36^ However, these cut‐offs were related to cardiovascular disease, hence no amendments were made for Asian participants.

Current WHO guidelines include dietary and exercise interventions during pregnancy to prevent excessive weight gain during this period.^39^ However, recommendations addressing sleep disorders during pregnancy are lacking.^5^ The prenatal period is ideal for healthy behavior change, as pregnant women regularly contact health professionals. Findings demonstrate that both BMI and sleep disorders need to be assessed at booking. Research is needed to find out if reducing gestational weight gain, particularly through dietary change, is of benefit to pregnant women with overweight or obesity at booking to reduce the presence of sleep disorders, minimize maternal and fetal consequences and improve the quality of life.^40^, ^41^ Healthcare professionals should advise and guide pregnant women with high BMI in early pregnancy. Their recommendations should include a healthy diet, exercise and sleep hygiene habits as preventive.^39^, ^42^, ^43^

The strengths of this study are the large sample size and the use of extensive and accurate data from different locations and ethnic groups in the multivariable analysis to prevent potential confounding bias. The multiethnic nature of the cohort also made it possible to analyze the differences more broadly.

A major limitation is that there was no assessment of the relationship between sleep and obesity was direct or related to the food choices and limited physical activity associated with obesity. This is an important area for future research. Further limitations include sleep parameters being self‐reported through a questionnaire survey conducted at booking rather than being objectively measured. This may have led to an overestimation of the occurrence of sleep disorders, with resultant recall or social desirability bias.^44^ Also, the questionnaires did not use any validated sleep assessment index, such as the PSQI scale,^31^ which measures the quality and patterns of sleep in adults. The questionnaire was based on some of the questions of the PSQI questionnaire and the Berlin questionnaire.^45^ Further studies using more accurate measures of sleep‐disordered breathing, such as the use of actigraphy that measures sleep duration and disruption, would be useful to minimize possible bias. Additionally, this study focused only on early pregnancy data; it remains unclear whether the relationship between BMI and sleep varies late in pregnancy. Finally, this study used a selected cohort of women at risk of GDM, so the results may not be generalizable to a wider population. Limited nutritional data were collected, and BMI was therefore used as a proxy for all its contributing factors.

In conclusion, this study in a large multiethnic cohort of pregnant women at risk of GDM revealed a significant relationship between severe obesity and sleep parameters, such as insufficient sleep and snoring. Women of both European and non‐European ethnicities were largely similar, except for a higher rate of experiencing insufficient sleep >5 days per month among European women. Further research is required to support the findings, investigate changes across different stages of pregnancy and ascertain the role of diet in sleep disturbances.

## AUTHOR CONTRIBUTIONS

Pamela Acosta Reyes and Jincy Immanuel performed statistical analysis, interpreted the data, and drafted the manuscript. David Simmons conceived the TOBOGM project and this analysis, and together with JI supervised PAR, interpreted the data, reviewed, and edited the draft and provided critical input to the manuscript. All authors read and approved the final manuscript. DS is the guarantor of this work and, as such, had full access to all the data in the study and takes responsibility for the integrity of the data and the accuracy of the data analysis.

## CONFLICT OF INTEREST STATEMENT

The authors declared no potential conflicts of interest with respect to the research, authorship and/or publication of this article.
